# Caregiver childhood abuse and neglect and children's cognitive and social-emotional development: The role of parenting practices

**DOI:** 10.1016/j.ssmph.2026.101988

**Published:** 2026-10-15

**Authors:** Qiannan Song, Hao Gong, Mingchuan Liu, Na Qiao, Ai Yue, Yaojiang Shi

**Affiliations:** a Center for Experimental Economics in Education (CEEE), Faculty of Education, Shaanxi Normal University, Xi'an, 710119, China; b School of Preschool Education, Xi'an University, Xi'an, 710065, China

**Keywords:** Childhood abuse and neglect, Child development, Parenting practices, Grandparental caregiving, Rural China

## Abstract

Longitudinal evidence on how caregivers' own childhood abuse and neglect (CA&N) is associated with children's development remains limited in low- and middle-income countries, where grandparents often serve as primary caregivers. Using five waves (2015–2024) of data from 1243 children in rural western China, we examined associations between caregiver CA&N and children's cognitive and social-emotional development, as well as statistical mediation through parenting practices, using mixed-effects models and bootstrap mediation analyses. Children whose caregivers reported CA&N showed persistently lower cognitive performance and greater social-emotional difficulties, with cognitive disparities widening over time. Physical neglect was associated with both developmental domains, whereas emotional neglect was primarily associated with social-emotional outcomes, with suggestive evidence that cognitive disparities emerged gradually over time. Statistical mediation was limited mainly to social-emotional outcomes, where indirect associations ran chiefly through neglectful parenting; there was little evidence of mediation for cognitive outcomes. Associations did not differ significantly between parental and grandparental caregivers, although point estimates were larger for parents. Neglectful parenting may be one route through which caregiver CA&N is linked to children's social-emotional difficulties, which points to the value of supporting caregivers with a history of CA&N.

## Highlights

• Caregivers' childhood abuse and neglect was associated with poorer child development.
• Cognitive gaps widened with age, whereas social-emotional gaps remained stable.
• Physical neglect related to both domains; emotional neglect mainly social-emotional.
• Mediation was limited, mainly via neglectful parenting for social-emotional outcomes.
• Associations did not differ significantly between parent and grandparent caregivers.

## 1 Introduction

Childhood abuse and neglect (CA&N)—including physical, emotional and sexual abuse, as well as physical and emotional neglect—refers to acts or omissions by parents or caregivers that harm a child's health, development, or dignity (Badenes-Ribera et al., 2024; He et al., 2019). Conceptually, CA&N constitutes a core component of the broader framework of adverse childhood experiences (ACEs). CA&N remains highly prevalent worldwide: a review of meta-analyses estimated prevalence rates of 12.7% for sexual abuse, 22.6% for physical abuse, 36.3% for emotional abuse, 16.3% for physical neglect, and 18.4% *for* emotional neglect (Stoltenborgh et al., 2015). ACEs are also common in China. A national survey of 9378 adults found that 27% reported at least one ACE, with neglect among the most commonly reported forms (Xu et al., 2024), and regional studies of mothers report even higher prevalence, reaching 69.7% in Anhui and 85.8% in Sichuan (Chen et al., 2023; Wang et al., 2022). Such experiences can have consequences extending well beyond childhood: early exposure to CA&N may disrupt neurodevelopment, immune function, and stress-regulation systems, and is associated with increased vulnerability to mental and physical illness across the life course (Dye, 2018; McEwen & Gregerson, 2019; Sales et al., 2024).

Beyond its consequences for individuals who experience it directly, a growing literature suggests that the risks associated with CA&N may extend across generations (Racine et al., 2025; van IJzendoorn et al., 2020; Zhang, Mersky, Gruber, & Kim, 2023). Children of caregivers—particularly parents—with histories of CA&N have been found to experience greater cognitive, social-emotional, and behavioral difficulties (Folger et al., 2018; Madigan et al., 2025; Racine et al., 2018; Zhang et al., 2024; Zhu et al., 2025). In China, cross-sectional studies have similarly linked maternal CA&N to lower quality of life and more behavioral problems among preschool-aged children (Chen et al., 2023; Luo et al., 2023). The processes underlying these intergenerational associations are likely multifaceted rather than attributable to a single mechanism (Zhang et al., 2024; Zhang, Mersky, Gruber, & Kim, 2023).

Several pathways may link caregivers' CA&N to their children's development. Caregiver mental health is one well-established pathway: CA&N is associated with later depression, psychological distress, and emotion-regulation difficulties, which are in turn associated with children's developmental outcomes (Swords et al., 2024; Zhang, Mersky, Gruber, & Kim, 2023; Zhang, Mersky, & Lee, 2023; Zhu et al., 2025). Socioeconomic disadvantage represents another potential pathway, while family functioning and social support have also been examined as mediators of intergenerational associations (Zhang, Mersky, Gruber, & Kim, 2023). We focus on parenting practices for three reasons. First, parenting represents a proximal pathway embedded in children's daily experiences, and caregiver mental health and socioeconomic strain are thought to affect children partly through everyday caregiving (Conger & Donnellan, 2007; Narayan et al., 2021). Second, stimulation, responsiveness, and neglectful parenting are relevant to both cognitive and social-emotional development and can be examined in the same way for parents and grandparents. Third, parenting is modifiable and is the direct target of many early childhood interventions (Narayan et al., 2021; Weistra et al., 2025). We do not assume that parenting is the only or the dominant pathway. Empirically, caregivers with histories of CA&N are more likely to engage in harsh or maladaptive parenting, show fewer positive behaviors, and exhibit neglectful responses (Herbert et al., 2023; Lotto et al., 2023; Ongilio et al., 2023; Plant et al., 2017; Savage et al., 2019; Weistra et al., 2025). Attachment theory offers one explanation: caregivers' early relational experiences shape their internal working models of caregiving and their capacity to read children's cues and respond sensitively (Bowlby, 1973; Lieberman et al., 2020; Pryce, 1995). CA&N may therefore undermine sensitive caregiving and increase neglectful or inconsistent parenting (Main & Hesse, 1990; Weistra et al., 2025). Positive and neglectful parenting may thus be complementary pathways linking caregiver CA&N to child development.

Despite growing international evidence on the intergenerational correlates of CA&N, several important questions remain. First, relatively little is known about how developmental disparities associated with caregiver CA&N unfold as children age—whether they emerge early, persist, or widen over time. Several longitudinal studies have documented associations between parental CA&N and offspring development, but this evidence has been generated largely in high-income Western countries (Madigan et al., 2025; Plant et al., 2017; Racine et al., 2018; Zhang, Mersky, Gruber, & Kim, 2023; Zhang, Mersky, & Lee, 2023). Evidence from low- and middle-income countries (LMICs) and non-Western settings remains limited (Zhang, Mersky, Gruber, & Kim, 2023), and studies in China have relied largely on cross-sectional designs (Luo et al., 2023; Wang et al., 2022). Second, existing research has concentrated primarily on children's social-emotional and behavioral outcomes (Luo et al., 2023; Wang et al., 2022; Zhang, Mersky, & Lee, 2023; Zhu et al., 2025). A scoping review of 68 intergenerational studies found that internalizing and externalizing problems were the most frequently examined outcomes, whereas only two studies examined academic performance, and cognitive outcomes were assessed mainly as one component of infant and toddler development (Zhang, Mersky, Gruber, & Kim, 2023). Cognitive development—a key determinant of educational attainment and long-term human capital formation—has therefore received comparatively less attention. Third, although caregivers' CA&N has been associated with both reduced positive parenting and increased negative or neglectful parenting (Greene et al., 2020; Savage et al., 2019; Weistra et al., 2025), these dimensions have usually been examined separately. Their relative contributions as complementary pathways therefore remain unclear, particularly in multigenerational caregiving contexts and in LMIC settings. Fourth, intergenerational research on CA&N has focused predominantly on mothers (Lotto et al., 2023, Ongilio et al., 2023, Zhang et al., 2023), and it remains unclear whether similar associations hold when other caregivers assume primary caregiving responsibility.

To address these questions, the present study draws on five waves of longitudinal data collected from 2015 to 2024 in rural western China, following children from infancy to middle childhood. In this setting, parental labor migration means that many children are cared for mainly by grandparents (Luo et al., 2008; Tan et al., 2010; Yue et al., 2020). These grandparents belong to a generation with distinct historical and socioeconomic experiences, including greater exposure to famine-related deprivation and other forms of material hardship (Fan & Qian, 2015), conditions that are established risk factors for CA&N (van IJzendoorn et al., 2020), and they remain understudied in intergenerational research on CA&N. This setting and design offer several advantages. Repeated age-appropriate assessments allow us to trace children's cognitive and social-emotional trajectories over nearly a decade. Measures of both positive and neglectful parenting allow these pathways to be examined within the same study. The prevalence of grandparental caregiving also yields a substantial sample of both parental and grandparental primary caregivers in a resource-constrained LMIC context where caregiver CA&N is common. Specifically, we examine (1) whether primary caregivers' own CA&N is associated with children's cognitive and social-emotional development over time; (2) whether positive and neglectful parenting statistically account for part of these associations; and (3) whether these associations differ between parental and grandparental caregivers. Given the observational nature of the data, our aim is to characterize longitudinal associations and statistical mediation rather than to estimate causal effects.

## 2 Methods

### 2.1 Sample and design

This study uses longitudinal data collected between 2015 and 2024 from a randomized controlled trial of a center-based parenting program implemented in rural western China. The trial was conducted in 20 low-income counties of the Qinba Mountain region and aimed to evaluate the impacts on early childhood development (Yue et al., 2024). Five household survey waves have been completed, comprising 5575 child-wave observations from 1243 children in total: 1223 at baseline (Wave 0), 1053 at Wave 1, 988 at Wave 2, 1126 at Wave 3, and 1185 at Wave 4.

In this study, the primary caregiver was defined as the individual primarily responsible for the child's daily care and routine parenting activities. In rural China, parental labor migration is common and grandparents frequently assume primary caregiving roles in left-behind households.

Information on the primary caregiver's CA&N was collected in Wave 4 using a retrospective instrument referring to experiences that occurred before the caregiver turned 16. Although reported at the final wave, these experiences occurred during the caregiver's own childhood, decades before the parenting practices measured in Waves 3–4 and the child developmental outcomes assessed throughout the study period.

The Wave 4 caregiver was defined as the reference caregiver, and their CA&N information was linked retrospectively to children's development outcomes across all survey waves. The primary analytic sample included children whose Wave 4 reference caregiver had valid CA&N data and who contributed at least one valid developmental assessment, regardless of primary caregiver changes over time. To address potential exposure misclassification arising from changes in primary caregivers over time, we additionally defined a consistent-caregiver subsample restricted to children whose primary caregiver remained the same from baseline through Wave 4. This subsample included 500 children contributing 2227 child-wave observations (Wave 0: 491; Wave 1: 414; Wave 2: 378; Wave 3: 444; Wave 4: 500), as shown in Fig. S1.

At each wave, trained enumerators conducted face-to-face interviews with caregivers and administered standardized assessments of child development. Children were aged 4–28 months at baseline (Wave 0), 17–40 months at Wave 1, 29–53 months at Wave 2, 51–83 months at Wave 3, and 90–125 months at Wave 4. This design captures developmental outcomes from infancy to middle childhood.

### **2.2** Measures

#### 2.2.1 Cognitive and social-emotional development

Children's cognition was assessed from 4 months to 10 years of age with age-appropriate standardized tests: the Bayley Scales of Infant and Toddler Development, Third Edition (BSID-III; Waves 0–2), the Wechsler Preschool and Primary Scale of Intelligence, Fourth Edition (WPPSI-IV; Wave 3), and the Wechsler Intelligence Scale for Children, Fourth Edition (WISC-IV; Wave 4). All were adapted for China and scores were standardized for comparability. Social-emotional skills were measured with the Ages & Stages Questionnaires: Social-Emotional (ASQ:SE) in Waves 0–2 (<66 months) and the Chinese version of the Strengths and Difficulties Questionnaire (SDQ) in Waves 3–4 (≥66 months) (Bian et al., 2021; Du et al., 2006). For both tools, standardized and reverse-coded scores were used, with higher values indicating better social-emotional development. Cognitive delay at baseline was defined as a BSID-III cognitive composite score more than one standard deviation below the mean reported for healthy reference populations (i.e., <95.4; reference mean = 105, SD = 9.6), following previous studies in rural China (Sylvia et al., 2022; Wang et al., 2019). Social-emotional delay at baseline was defined as an ASQ:SE total score above the age-specific cutoff specified in the ASQ:SE User's Guide (Squires et al., 2002), consistent with previous studies in rural China (Wang et al., 2024).

#### 2.2.2 Caregiver CA&N

Caregiver CA&N was assessed in Wave 4 using the 28-item Childhood Trauma Questionnaire-Short Form (CTQ-SF) (Bernstein et al., 2003). The Chinese CTQ-SF has demonstrated strong reliability and validity (He et al., 2019). The CTQ-SF includes five subscales—emotional abuse (EA), physical abuse (PA), sexual abuse (SA), emotional neglect (EN), and physical neglect (PN). Each subscale comprises 5 items rated on a five-point Likert scale, which were summed to yield subscale scores ranging from 5 to 25. Following the moderate-to-severe cutoffs in the Childhood Trauma Questionnaire manual (Bernstein & Fink, 1998), the subscales were dichotomized at EN ≥ 15, PN ≥ 10, EA ≥ 13, PA ≥ 10, and SA ≥ 8. A caregiver was classified as exposed to a subtype when the corresponding score met or exceeded its cutoff. The primary CA&N indicator was coded as 1 for exposure to at least one subtype and 0 for none. The composite indicator therefore covers all five subscales. Because EA, PA, and SA were each rare in this sample (Table 1), subtype-specific analyses were limited to PN and EN.

**Table 1:** Descriptive statistics of the study sample.

Variables	Full sample	Parent	Grandparent	p-value
**Panel A. Sample characteristics (Wave 0)**				
Child characteristics				
Sex (Male = 1)	0.52 (0.50)	0.51 (0.50)	0.54 (0.50)	0.468
Only child (Yes = 1)	0.46 (0.50)	0.39 (0.49)	0.65 (0.48)	< 0.001
Left-behind child (ever during Waves 0–4; Yes = 1)	0.45 (0.50)	0.33 (0.47)	0.80 (0.40)	< 0.001
Caregiver characteristics				
Primary caregiver is mother (Yes = 1)	0.72 (0.45)	0.98 (0.13)	NA	NA
Maternal age at delivery	26.52 (4.90)	27.15 (5.03)	24.69 (3.93)	< 0.001
Caregiver education (Junior high & above = 1)	0.14 (0.35)	0.18 (0.38)	0.04 (0.20)	< 0.001
Household characteristics				
Household assets (High = 1)	0.50 (0.50)	0.52 (0.50)	0.45 (0.50)	0.033
Baseline child outcomes				
Cognitive delay (Yes = 1)	0.54 (0.50)	0.54 (0.50)	0.55 (0.50)	0.696
Social-emotional delay (Yes = 1)	0.40 (0.49)	0.40 (0.49)	0.41 (0.49)	0.674
N	1223	898	321	
**Panel B. Endline evaluation (Wave 4)**				
Number of Caregiver CA&N subtypes				
0	0.50 (0.50)	0.58 (0.49)	0.33 (0.47)	< 0.001
1	0.32 (0.47)	0.28 (0.45)	0.40 (0.49)	< 0.001
2	0.14 (0.34)	0.10 (0.30)	0.21 (0.41)	< 0.001
≥3	0.04 (0.20)	0.03 (0.17)	0.06 (0.24)	0.021
Caregiver CA&N subtypes				
CA&N (Yes = 1)	0.50 (0.50)	0.42 (0.49)	0.67 (0.47)	< 0.001
Physical neglect (Yes = 1)	0.46 (0.50)	0.37 (0.48)	0.65 (0.48)	< 0.001
Emotional neglect (Yes = 1)	0.18 (0.39)	0.16 (0.36)	0.24 (0.43)	0.001
Physical abuse (Yes = 1)	0.03 (0.16)	0.02 (0.14)	0.04 (0.19)	0.132
Emotional abuse (Yes = 1)	0.02 (0.14)	0.01 (0.12)	0.03 (0.17)	0.080
Sexual abuse (Yes = 1)	0.05 (0.22)	0.03 (0.17)	0.09 (0.28)	< 0.001
N	1185	796	386	

*Note.* Data are mean (SD); CA&N, childhood abuse and neglect; NA, not applicable. Caregiver type refers to the primary caregiver at the corresponding wave (Wave 0 in Panel A and Wave 4 in Panel B). Variables in Panel A were measured at Wave 0, except left-behind status. Cognitive delay was defined as a BSID-III cognitive composite score below 95.4, and social-emotional delay as an ASQ:SE total score above the age-specific cutoff. The sum of the parent and grandparent subsamples does not equal the total sample size because a small proportion of children were cared for by other caregiver types (e.g., relatives or non-kin). Group differences between parent and grandparent subsamples were tested using chi-squared tests for categorical variables and two-sample t-tests for continuous variables.

The cutoff-based specification was adopted because the thresholds represent clinically meaningful levels of CA&N exposure, enhance the interpretability of comparisons between exposed and unexposed caregivers, and facilitate comparability with previous epidemiological studies. Because dichotomization may discard information and reduce statistical power, we conducted robustness analyses using alternative CTQ-SF specifications, including the standardized total score and the number of CA&N subtypes exceeding clinical cutoffs. These analyses yielded broadly consistent findings (Table S1).

#### 2.2.3 Parenting practices

Parenting practices were measured in Waves 3 and 4. Positive parenting was assessed by caregiver-reported activities (reading, storytelling, singing, playing, and discussing experiences), aggregated into standardized scores, with higher values indicating more engagement. Neglectful parenting was measured with the Rural Child Neglect Assessment Scale, a tool validated in China covering six domains (physical, emotional, educational, medical, safety, social) (Pan et al., 2014). Wave 3 included 57 items and Wave 4 included 69; higher total scores reflected more neglectful parenting of the study child.

#### 2.2.4 Covariates

Unless otherwise noted, all models adjusted for child sex, birth order, left-behind status, whether the primary caregiver is the child's mother, maternal age at delivery, primary caregiver's education (junior high school or above), household assets, and original trial arm. Because the data analyzed in this study were collected within a cluster-randomized controlled trial of a parenting program (Yue et al., 2024), all models included the randomized trial assignment (“original trial arm” in the tables) as a covariate to account for the original study design. Left-behind status was coded 1 if both parents had migrated for work during the same survey wave in at least one of the five waves, and 0 otherwise. Whether the primary caregiver was the child's mother and the primary caregiver's education referred to the primary caregiver at each wave. Household assets were also measured at each wave, using ten binary indicators of household ownership of durable goods and amenities (e.g., flush toilet, water heater, refrigerator, washing machine, and motor vehicle). These indicators were combined using principal component analysis to generate a composite asset score, which was then dichotomized at the wave-specific sample mean (1 = at or above the mean, 0 = below). Full-sample longitudinal models additionally adjusted for an indicator of primary caregiver change across waves (Section 2.1).

### 2.3 Statistical analysis

We used multilevel mixed-effects regression models to estimate longitudinal associations between primary caregiver CA&N and children's cognitive and social-emotional outcomes across the five survey waves (Laird & Ware, 1982; Singer & Willett, 2003). Repeated developmental assessments (Level 1) were nested within children (Level 2); these models account for the correlation among repeated observations from the same child and accommodate unbalanced data under the missing-at-random assumption. Caregiver CA&N-by-wave interaction terms allowed us to examine whether the association between caregiver CA&N and child development changed across survey waves.

Because only one study child was enrolled from each household, the household-level clustering was not separately modeled. Village-level intraclass correlation coefficients were small (0.03 for cognitive outcomes and 0.01 for social-emotional outcomes). We therefore specified the more parsimonious two-level model as the primary specification and examined village-level dependence in sensitivity analyses. Specifically, we fitted three-level mixed-effects models with village-level random intercepts and models with standard errors clustered at the village level. These alternative specifications produced results that were highly consistent with those from the primary analyses (Table S2).

The general model was specified as:

(1) $$Y_{it}=\beta_{0}+\beta_{1}{c\_wave}_{it}+\beta_{2}{c\_wave}_{it}^{2}+\beta_{3}{CAN}_{i}+\beta_{4}{CAN}_{i}\times {c\_wave}_{it}+\gamma X_{i}+u_{0i}+u_{1i}{c\_wave}_{it}+\varepsilon_{it}$$

where $Y_{it}$ denotes the standardized cognitive or social-emotional development score of child i at wave t. ${CAN}_{i}$ is a binary indicator for whether the primary caregiver experienced CA&N in her or his own childhood. ${c\_wave}_{it}$ is the mean-centered survey wave, with a quadratic term (${c\_wave}_{it}^{2}$) allowing for non-linear patterns over time. $X_{i}$ is the vector of covariates defined in Section 2.2.4. The child-level random intercept and slope ($u_{0i}$, $u_{1i}$) are assumed bivariate normal, and $\varepsilon_{it}$ is the residual, assumed normally distributed and independent of the random effects. With the survey wave mean-centered, the coefficient on ${CAN}_{i}$ represents the exposure-group difference at the average wave. The random-effects structure was selected by comparing model fit (Akaike and Bayesian information criteria, AIC and BIC) across preliminary specifications; the quadratic wave term and the CA&N × wave interaction were specified in line with our research questions and evidence of non-linear developmental trajectories, and were retained in the final model.

We conducted several sensitivity analyses to assess the robustness of the findings. First, we re-estimated the primary models in the consistent-caregiver subsample defined in Section 2.1, using the same centered wave variable. Second, to examine whether the findings were influenced by participation in the parenting intervention, we repeated the analyses among control-group families only (Table S3). Third, we applied propensity score matching to improve covariate balance between caregivers with and without CA&N exposure (Table S4). Additional robustness analyses examined alternative specifications of caregiver CA&N (Table S1) and alternative approaches to accounting for village-level clustering (Table S2).

To improve comparability between exposed and unexposed groups on observed covariates, we estimated propensity scores using logistic regression, with caregiver CA&N as the dependent variable and child sex, birth order, left-behind status, maternal age at delivery, caregiver education, household assets, caregiver-change status, and original trial arm as predictors (Austin, 2011; Rosenbaum & Rubin, 1983). We then applied one-to-one nearest-neighbor matching with replacement on the propensity score using a caliper of 0.05 and re-estimated the mixed-effects models in the matched sample (Table S4). Covariate balance was evaluated by comparing propensity score distributions and covariate-specific standardized mean differences before and after matching. After matching, all absolute standardized mean differences were below the conventional 0.10 threshold (Fig. S2 and S3).

To explore potential pathways underlying the observed associations, we conducted statistical mediation analyses to examine whether parenting practices accounted for part of the associations between caregiver CA&N and children's cognitive and social-emotional outcomes. Indirect associations were estimated using the product-of-coefficients approach (MacKinnon et al., 2002), adjusting for the covariates defined in Section 2.2.4, an indicator of primary caregiver change, and the developmental outcome measured at the preceding wave. Because the sampling distribution of the product of coefficients is typically non-normal, inference was based on bias-corrected 95% confidence intervals (CIs) from 1000 non-parametric bootstrap replications (Preacher & Hayes, 2004, 2008). Analyses were implemented with the sgmediation2 module in Stata (Mize, 2022).

Finally, we examined heterogeneity by caregiver type. Mixed-effects models were estimated separately for children cared for by parents and by grandparents according to the Wave 4 reference caregiver type, adjusting for child- and family-level covariates. Differences between parent- and grandparent-specific coefficients were evaluated with tests of coefficient equality.

## 3 Results

### 3.1 Demographic characteristics

Table 1 (Panel A) presents the baseline characteristics of the study sample. Among children, 52% were male, 46% were only children, and 45% were classified as left-behind children. Baseline characteristics differed according to primary caregiver type. Compared with children cared for by parents, those cared for by grandparents were more likely to be only children (65% vs. 39%) and left-behind (80% vs. 33%).

Among primary caregivers, 72% were mothers. Overall educational attainment was low, with only 14% having completed junior high school or above. Attainment was lower among grandparents, of whom only 4% had completed junior high school or above, compared with 18% of parents. Mothers of children primarily cared for by grandparents were younger at delivery than those of children cared for by parents (mean age 25 vs. 27 years).

At baseline, 54% of children exhibited cognitive delays and 40% showed social-emotional delays. The prevalence of both cognitive and social-emotional delays was similar across caregiver types (cognitive: 55% vs. 54%; social-emotional delays: 41% vs. 40%).

At Wave 4 (Panel B), 50% of caregivers reported no exposure to CA&N, while 32% reported one type, 14% reported two types, and 4% reported three or more. PN was the most prevalent CA&N subtype (46%), followed by EN (18%), whereas abuse subtypes were relatively uncommon (2–5%). Grandparents reported more CA&N than parents overall (67% vs. 42%) and for PN, EN, and SA, whereas differences in PA and EA were not statistically significant.

### 3.2 Associations between caregiver CA&N and children's developmental outcomes

Table 2 and Fig. S4 present the longitudinal associations between caregiver CA&N and children's standardized cognitive and social-emotional scores. Analyses were conducted in the full sample (5575 child-wave observations) and replicated in a consistent-caregiver subsample (2227 child-wave observations).

**Table 2:** Longitudinal associations between caregiver CA&N and children's cognitive and social-emotional outcomes (from 4 months to 10 years of age).

Variables	Standardized cognitive scores						Standardized social-emotional scores					
	Full sample (5575 child-wave observations)			Consistent-caregiver subsample (2227 child-wave observations)			Full sample (5575 child-wave observations)			Consistent-caregiver subsample (2227 child-wave observations)		
	β (SE)	p-value	95% CI	β (SE)	p-value	95% CI	β (SE)	p-value	95% CI	β (SE)	p-value	95% CI
Caregiver CA&N (=1; Ref = 0)	−0.12** (0.04)	0.002	−0.20 to −0.04	−0.13* (0.06)	0.039	−0.26 to −0.01	−0.21*** (0.04)	< 0.001	−0.28 to −0.14	−0.36*** (0.06)	< 0.001	−0.48 to −0.24
Wave (centered)	0.02* (0.01)	0.046	0.00 to 0.05	0.02 (0.02)	0.235	−0.01 to 0.06	0.01 (0.01)	0.641	−0.02 to 0.03	0.02 (0.02)	0.302	−0.02 to 0.06
Wave (centered)^2^	−0.01 (0.01)	0.087	−0.02 to 0.00	−0.02 (0.01)	0.055	−0.04 to 0.00	0.04*** (0.01)	< 0.001	0.02 to 0.05	0.03*** (0.01)	< 0.001	0.01 to 0.05
Caregiver CA&N × Wave	−0.05** (0.02)	0.007	−0.08 to −0.01	−0.07** (0.03)	0.009	−0.12 to −0.02	−0.02 (0.02)	0.368	−0.05 to 0.02	−0.03 (0.03)	0.226	−0.09 to 0.02
Primary caregiver changed (=1)	0.09* (0.04)	0.026	0.01 to 0.18				0.14*** (0.04)	< 0.001	0.06 to 0.21			
Constant	−0.17 (0.15)	0.273	−0.47 to 0.13	−0.01 (0.26)	0.970	−0.53 to 0.51	−0.04 (0.14)	0.762	−0.32 to 0.23	0.07 (0.25)	0.783	−0.42 to 0.56

*Note.* Standard errors in parentheses. Values of 1 indicate caregiver CA&N exposure, and 0 indicate no exposure. CA&N, childhood abuse and neglect; Ref, reference group; β, standardized beta; CI, confidence interval; Wave (centered), survey wave centered at the full-sample mean in both samples. Models control for child sex, birth order, left-behind status, whether the caregiver is the mother, maternal age at delivery, caregiver education, household assets, and original trial arm; full-sample models additionally adjust for primary caregiver change. Consistent-caregiver subsample includes children whose primary caregiver remained the same across all five waves. Primary caregiver changed (1 = changed at least once across waves, 0 = no change). *p < 0.05, **p < 0.01, ***p < 0.001.

For cognitive outcomes, caregiver CA&N was associated with lower cognitive scores in both the full sample (β = −0.12, p = 0.002) and the consistent-caregiver subsample (β = −0.13, p = 0.039). A significant negative CA&N × wave interaction indicated that cognitive disparities widened over time; the quadratic wave term was not significant.

For social-emotional outcomes, caregiver CA&N was associated with poorer social-emotional development in both the full sample (β = −0.21, p < 0.001) and the consistent-caregiver subsample (β = −0.36, p < 0.001). The CA&N × wave interaction was not significant in either sample (β = −0.02, p = 0.368; β = −0.03, p = 0.226), indicating that the social-emotional gap remained stable across waves.

Findings were similar in analyses restricted to control-group families (Table S3) and after propensity score matching (Table S4).

### 3.3 Associations between specific caregiver CA&N subtypes and children's developmental outcomes

Table 3 and Fig. S5 present associations between caregiver CA&N subtypes and children's cognitive and social-emotional outcomes.

**Table 3:** Longitudinal associations between specific caregiver CA&N subtypes and children's cognitive and social-emotional outcomes (from 4 months to 10 years of age).

Variables	Standardized cognitive scores						Standardized social-emotional scores					
	Full sample (5575 child-wave observations)			Consistent-caregiver subsample (2227 child-wave observations)			Full sample (5575 child-wave observations)			Consistent-caregiver subsample (2227 child-wave observations)		
	β (SE)	p-value	95% CI	β (SE)	p-value	95% CI	β (SE)	p-value	95% CI	β (SE)	p-value	95% CI
**Panel A. Physical neglect (PN)**												
Caregiver PN (=1; Ref = 0)	−0.13*** (0.04)	< 0.001	−0.21 to −0.05	−0.14* (0.07)	0.027	−0.27 to −0.02	−0.20*** (0.04)	< 0.001	−0.27 to −0.13	−0.37*** (0.06)	< 0.001	−0.49 to −0.25
Wave (centered)	0.02 (0.01)	0.088	0.00 to 0.04	0.02 (0.02)	0.368	−0.02 to 0.05	0.00 (0.01)	0.819	−0.02 to 0.03	0.02 (0.02)	0.290	−0.02 to 0.06
Wave (centered)^2^	−0.01 (0.01)	0.086	−0.02 to 0.00	−0.02 (0.01)	0.055	−0.04 to 0.00	0.04*** (0.01)	< 0.001	0.02 to 0.05	0.03*** (0.01)	< 0.001	0.01 to 0.05
PN × Wave	−0.04* (0.02)	0.016	−0.07 to −0.01	−0.06* (0.03)	0.022	−0.12 to −0.01	−0.01 (0.02)	0.544	−0.04 to 0.02	−0.04 (0.03)	0.192	−0.09 to 0.02
Primary caregiver changed (=1)	0.09* (0.04)	0.028	0.01 to 0.17				0.13*** (0.04)	< 0.001	0.06 to 0.21			
Constant	−0.17 (0.15)	0.265	−0.47 to 0.13	−0.02 (0.26)	0.934	−0.54 to 0.49	−0.06 (0.14)	0.657	−0.34 to 0.21	0.03 (0.25)	0.911	−0.46 to 0.52
**Panel B. Emotional neglect (EN)**												
Caregiver EN (=1; Ref = 0)	−0.05 (0.05)	0.302	−0.15 to 0.05	−0.10 (0.08)	0.215	−0.26 to 0.06	−0.27*** (0.04)	< 0.001	−0.36 to −0.18	−0.34*** (0.08)	< 0.001	−0.49 to −0.19
Wave (centered)	0.01 (0.01)	0.354	−0.01 to 0.03	0.01 (0.02)	0.635	−0.02 to 0.04	0.00 (0.01)	0.605	−0.01 to 0.02	0.02 (0.02)	0.339	−0.02 to 0.05
Wave (centered)^2^	−0.01 (0.01)	0.081	−0.02 to 0.00	−0.02 (0.01)	0.053	−0.04 to 0.00	0.04*** (0.01)	< 0.001	0.02 to 0.05	0.03*** (0.01)	< 0.001	0.01 to 0.05
EN × Wave	−0.04 (0.02)	0.052	−0.09 to 0.00	−0.09** (0.03)	0.007	−0.16 to −0.03	−0.04 (0.02)	0.093	−0.08 to 0.01	−0.06 (0.04)	0.114	−0.13 to 0.01
Primary caregiver changed (=1)	0.09* (0.04)	0.036	0.01 to 0.17				0.12** (0.04)	0.001	0.05 to 0.20			
Constant	−0.23 (0.15)	0.129	−0.53 to 0.07	−0.05 (0.26)	0.844	−0.57 to 0.46	−0.11 (0.14)	0.407	−0.39 to 0.16	−0.03 (0.25)	0.918	−0.52 to 0.47

*Note.* Standard errors in parentheses. Values of 1 indicate caregiver PN (Panel A) or EN (Panel B) exposure, and 0 indicate no exposure. PN, physical neglect; EN, emotional neglect; Ref, reference group; β, standardized beta; CI, confidence interval; Wave (centered), survey wave centered at the full-sample mean in both samples. Models control for child sex, birth order, left-behind status, whether the caregiver is the mother, maternal age at delivery, caregiver education, household assets, and original trial arm; full-sample models additionally adjust for primary caregiver change. Consistent-caregiver subsample includes children whose primary caregiver remained the same across all five waves. Primary caregiver changed (1 = changed at least once across waves, 0 = no change). *p < 0.05, **p < 0.01, ***p < 0.001.

For cognitive outcomes, PN exposure was consistently associated with lower standardized cognitive scores in both the full sample (β = −0.13, p < 0.001) and the consistent-caregiver subsample (β = −0.14, p = 0.027). A significant PN × wave interaction indicated that these disparities widened over time (full sample: β = −0.04, p = 0.016; consistent-caregiver subsample: β = −0.06, p = 0.022); the quadratic wave terms were not significant. By contrast, EN was not significantly associated with cognitive scores at the average wave in either the full sample (β = −0.05, p = 0.302) or the consistent-caregiver subsample (β = −0.10, p = 0.215). However, the EN × wave interaction was negative and statistically significant in the consistent-caregiver subsample (β = −0.09, p = 0.007) and marginally significant in the full sample (β = −0.04, p = 0.052), suggesting that cognitive disparities associated with EN emerged gradually over time.

For social-emotional development, exposure to both PN and EN was associated with poorer outcomes, with associations larger in the consistent-caregiver subsample (PN: β = −0.20, p < 0.001 in the full sample and β = −0.37, p < 0.001 in the consistent-caregiver subsample; EN: β = −0.27, p < 0.001 and β = −0.34, p < 0.001, respectively). Neither the PN × wave nor the EN × wave interaction reached statistical significance, indicating that social-emotional disparities associated with PN and EN remained broadly stable across waves.

### 3.4 Statistical mediation through parenting practices in the associations between caregiver CA&N and children's developmental outcomes

Table 4 presents the estimated indirect associations of caregiver CA&N with children's developmental outcomes through positive and neglectful parenting, adjusted for covariates and for the same outcome at the preceding wave.

**Table 4:** Statistical mediation (indirect associations) via positive and neglectful parenting in the associations between caregiver CA&N and children's cognitive and social-emotional development.

Wave	Path	β	SE	95% CI (BC)
Cognitive development				
Panel A. Positive parenting as mediator				
Wave 3	CA&N → Parenting_W3→ Cognition_W3	−0.01	0.01	−0.02 to 0.00
Wave 4	CA&N → Parenting_W4→ Cognition_W4	−0.01	0.01	−0.02 to 0.00
Cross-wave	CA&N → Parenting_W3→ Cognition_W4	0.00	0.00	−0.01 to 0.01
Panel B. Neglectful parenting as mediator				
Wave 3	CA&N → Neglect_W3→ Cognition_W3	−0.01	0.01	−0.03 to 0.01
Wave 4	CA&N → Neglect_W4→ Cognition_W4	−0.02	0.02	−0.06 to 0.02
Cross-wave	CA&N → Neglect_W3→ Cognition_W4	−0.02	0.01	−0.04 to 0.00†
Social-emotional development				
Panel C. Positive parenting as mediator				
Wave 3	CA&N → Parenting_W3→ SocEmot_W3	−0.01	0.01	−0.02 to 0.00
Wave 4	CA&N → Parenting_W4→ SocEmot_W4	−0.03	0.01	−0.06 to −0.01†
Cross-wave	CA&N → Parenting_W3→ SocEmot_W4	0.00	0.00	−0.01 to 0.01
Panel D. Neglectful parenting as mediator				
Wave 3	CA&N → Neglect_W3→ SocEmot_W3	−0.07	0.02	−0.11 to −0.03†
Wave 4	CA&N → Neglect_W4→ SocEmot_W4	−0.15	0.03	−0.21 to −0.10†
Cross-wave	CA&N → Neglect_W3→ SocEmot_W4	−0.02	0.01	−0.04 to 0.00

*Note.* CA&N, childhood abuse and neglect; β, standardized beta; SE, standard error; CI, confidence interval; BC, bias-corrected; W3, Wave 3; W4, Wave 4; Parenting, positive parenting; Neglect, neglectful parenting; SocEmot, social-emotional development. Models control for child sex, birth order, left-behind status, whether the caregiver is the mother, maternal age at delivery, caregiver education, household assets, original trial arm, primary caregiver change, and the same outcome measured at the preceding wave (Wave 2 for Wave 3 models; Wave 3 for Wave 4 and cross-wave models). Indirect associations were estimated using the product-of-coefficients approach, and statistical inference was based on 95% bias-corrected confidence intervals from 1000 non-parametric bootstrap replications. † Interval excludes zero (before rounding).

For cognitive outcomes (Panels A and B), there was little evidence of indirect associations. The confidence intervals for positive parenting included zero in all three models (Wave 3: β = −0.01, 95% CI: −0.02 to 0.00; Wave 4: β = −0.01, 95% CI: −0.02 to 0.00; cross-wave: β = 0.00, 95% CI: −0.01 to 0.01). For neglectful parenting, the concurrent estimates at Wave 3 (β = −0.01, 95% CI: −0.03 to 0.01) and Wave 4 (β = −0.02, 95% CI: −0.06 to 0.02) also included zero. Only the cross-wave estimate, from neglectful parenting at Wave 3 to cognition at Wave 4, excluded zero, and it was small (β = −0.02, 95% CI: −0.04 to 0.00; the upper bound was below zero before rounding).

For social-emotional outcomes (Panels C and D), indirect associations through neglectful parenting were observed at Wave 3 (β = −0.07, 95% CI: −0.11 to −0.03) and Wave 4 (β = −0.15, 95% CI: −0.21 to −0.10), whereas the cross-wave estimate included zero (β = −0.02, 95% CI: −0.04 to 0.00). Through positive parenting, an indirect association was observed only at Wave 4 (β = −0.03, 95% CI: −0.06 to −0.01); the Wave 3 (β = −0.01, 95% CI: −0.02 to 0.00) and cross-wave estimates (β = 0.00, 95% CI: −0.01 to 0.01) included zero.

Overall, evidence of statistical mediation was limited mainly to social-emotional outcomes and came chiefly from neglectful parenting measured in the same wave as the outcome. Indirect associations through positive parenting were small, and there was little evidence of mediation for cognitive outcomes.

### 3.5 Heterogeneous associations by primary caregiver type

Table 5 explores whether associations between caregiver CA&N and child development outcomes differed by caregiver type (parents vs. grandparents), defined by the Wave 4 reference caregiver.

**Table 5:** Associations between caregiver CA&N and children's cognitive and social-emotional outcomes, by caregiver type.

Variables	Standardized cognitive scores			Standardized social-emotional scores		
	Parents (3611 child-wave observations)	Grandparents (1758 child-wave observations)	Difference	Parents (3611 child-wave observations)	Grandparents (1758 child-wave observations)	Difference
Caregiver CA&N (=1; Ref = 0)	−0.14** (0.05)	−0.06 (0.06)	−0.08, p = 0.320	−0.26*** (0.04)	−0.17* (0.07)	−0.09, p = 0.289
Wave (centered)	0.01 (0.01)	−0.02 (0.01)	0.03, p = 0.067	0.01 (0.01)	−0.03* (0.02)	0.04, p = 0.035
Wave (centered)^2^	−0.01 (0.01)	−0.01 (0.01)	0.00, p = 0.996	0.03*** (0.01)	0.05*** (0.01)	−0.01, p = 0.386
Constant	−0.13 (0.19)	−0.03 (0.26)		−0.10 (0.17)	0.29 (0.28)	

*Note*. Standard errors in parentheses. Values of 1 indicate caregiver CA&N exposure, and 0 indicate no exposure. CA&N, childhood abuse and neglect; Ref, reference group; Wave (centered), survey wave centered at the full-sample mean. Caregiver type is defined by the Wave 4 reference caregiver, whose CA&N was assessed, and all waves of a child are assigned to the same group. Models control for child sex, birth order, left-behind status, maternal age at delivery, caregiver education, household assets, and original trial arm. Difference = β(Parents) – β(Grandparents), tested with a z test for the equality of coefficients from independent subsamples. The sum of the parent and grandparent subsamples does not equal the total sample size because children whose Wave 4 caregiver was another relative or non-kin, and children with no Wave 4 observation in the analytic sample, are not included. *p < 0.05, **p < 0.01, ***p < 0.001.

For cognitive outcomes, parental CA&N was associated with lower scores (β = −0.14, p = 0.006), whereas the estimate for grandparental CA&N was smaller and not statistically significant (β = −0.06, p = 0.369); the two estimates did not differ significantly (p = 0.320).

For social-emotional outcomes, CA&N was associated with poorer development for both parental (β = −0.26, p < 0.001) and grandparental caregivers (β = −0.17, p = 0.014), and the difference between them was not statistically significant (p = 0.289). Point estimates were larger for parents on both outcomes, but neither difference was statistically significant, so these subgroup patterns should be interpreted as suggestive.

## 4 Discussion

Using five waves of longitudinal data from rural western China, this study examined how caregiver CA&N was associated with children's cognitive and social-emotional development across childhood and whether parenting practices represented potential indirect pathways. Three main findings emerged. First, caregiver CA&N was consistently associated with poorer cognitive and social-emotional development, with cognitive disparities widening over time and social-emotional differences remaining comparatively stable; subtype analyses further suggested somewhat different patterns for PN and EN. Second, statistical mediation was limited mainly to social-emotional outcomes, where indirect associations ran chiefly through neglectful parenting; there was little evidence of mediation for cognitive outcomes. Third, associations did not differ significantly between parental and grandparental caregivers, although point estimates were larger for parents.

The longitudinal associations observed in this study are consistent with previous evidence linking caregivers’ CA&N with developmental outcomes in the next generation (Racine et al., 2025). Our findings extend a literature that has been concentrated largely in high-income settings by showing that these associations persisted across repeated assessments from infancy through middle childhood in an LMIC context and by documenting associations with cognitive development, which has received comparatively less attention. Cognitive disparities widened as children aged, whereas social-emotional differences remained relatively stable. One possible interpretation is that cognitive development may be particularly sensitive to the cumulative influence of differences in stimulation, learning opportunities, and caregiving resources across childhood (Prime et al., 2023), although our mediation analyses provided little evidence that the parenting dimensions measured here accounted for the cognitive association. By contrast, social-emotional difficulties associated with caregiver CA&N may become established earlier and show greater continuity over time. Subtype analyses were broadly consistent with these domain-specific patterns: PN was associated with poorer outcomes in both developmental domains, whereas EN showed a clearer association with social-emotional development and less consistent evidence for cognitive development.

Parenting practices were the main family-level pathway examined in this study. Previous evidence links CA&N with later parenting difficulties (Greene et al., 2020; Savage et al., 2019; Weistra et al., 2025). After adjustment for covariates and for the outcome at the preceding wave, however, evidence of statistical mediation in our data was limited. Indirect associations were observed mainly for social-emotional outcomes and ran chiefly through neglectful parenting measured in the same wave as the outcome; an indirect association through positive parenting was observed only at Wave 4. For cognitive outcomes, only the cross-wave indirect association through neglectful parenting excluded zero, and it was small. One possible explanation for the more consistent role of neglectful parenting is that neglectful parenting may reflect broader deficiencies in the caregiving environment, including reduced sensitivity and responsiveness and fewer nurturing, supportive interactions, which may be particularly relevant to children's social-emotional development (Narayan et al., 2021; Weistra et al., 2025). Examining positive and neglectful parenting in the same study showed that the two dimensions did not contribute equally, which would not be apparent if they were examined separately. Because the present analyses were observational, these findings indicate statistical rather than causal mediation. Parenting should therefore be viewed as one potential pathway rather than a complete explanation; caregiver mental health—which, together with parenting, is among the most frequently examined psychosocial pathways in this literature (Zhang, Mersky, Gruber, & Kim, 2023)—as well as socioeconomic conditions and other family-level processes may also contribute.

We also examined whether associations differed between parental and grandparental caregivers, which is particularly relevant in rural China, where grandparents often serve as primary caregivers. Grandparents in our sample reported more CA&N than parents, which may partly reflect generational differences in the socioeconomic and caregiving contexts in which they grew up (Liu, 2025). Point estimates were larger for parental than for grandparental CA&N for both cognitive and social-emotional outcomes, and the association with cognition was statistically significant only among parents. The formal between-group comparisons, however, did not reach statistical significance for either outcome. These patterns should therefore be treated as hypothesis-generating rather than as evidence of established caregiver-type heterogeneity. Differences in socioeconomic histories, educational opportunities, and caregiving circumstances may contribute to the observed patterns (Bai et al., 2019, 2021), but larger studies with direct measurement of the relevant pathways are needed.

These findings also have potential implications for intervention. Our mediation results give only limited support for parenting as a pathway, and mainly for neglectful parenting and social-emotional outcomes. With that caveat, programs that reduce neglectful parenting while strengthening responsive and stimulating caregiving may help address the social-emotional difficulties associated with caregiver CA&N (Narayan et al., 2021; Weistra et al., 2025). Approaches that recognize caregivers’ own histories of CA&N while providing parenting support may be particularly relevant. In rural China and other settings where grandparents frequently assume primary caregiving responsibilities, such efforts may also need to include grandparental caregivers rather than focusing exclusively on mothers.

This study has several strengths, including five waves of repeated developmental assessments spanning infancy through middle childhood, coverage of both cognitive and social-emotional domains, inclusion of both parental and grandparental primary caregivers, and extensive robustness analyses using alternative samples and exposure specifications. The repeated-measures design also allowed us to identify developmental patterns, such as the widening cognitive disparity, that would not be visible in single-wave analyses.

Several limitations should be acknowledged. First, caregivers' CA&N was assessed retrospectively and may therefore be subject to recall error and reporting bias. Prior evidence suggests that retrospective reports more often under- than over-identify childhood adversity (Hardt & Rutter, 2004); if such error is non-differential, it would tend to attenuate the estimated associations, although differential reporting cannot be ruled out. In addition, the primary caregiver identified at Wave 4 served as the reference caregiver whose childhood CA&N was linked to children's developmental outcomes measured at earlier waves. Although analyses restricted to children with stable primary caregivers and models adjusting for caregiver change yielded similar results, changes in caregiving arrangements may still weaken the correspondence between the measured caregiver exposure and children's earlier caregiving environments.

Second, the observational design limits causal interpretation. Adjustment for observed covariates and propensity score matching improved comparability between exposed and unexposed caregivers but cannot eliminate unmeasured confounding. The mediation analyses are subject to the same limitation and therefore identify indirect associations rather than causal mechanisms. In addition, most of the indirect associations that excluded zero were concurrent, with the mediator and the outcome measured in the same wave, so their temporal order cannot be established. Caregiver mental health, another well-established intergenerational pathway, was not examined as a mediator in the present study and should be considered jointly with parenting in future research.

Third, participants were originally recruited through a cluster-randomized parenting intervention, which may limit generalizability beyond the study population. However, analyses restricted to households in the original control group yielded substantively similar findings, suggesting that the observed associations were unlikely to be driven by intervention exposure.

Finally, several measurement limitations should be noted. The parenting measures did not capture the full range of potentially relevant caregiving behaviors, such as harsh discipline or coercive control. The primary CA&N measure relied on clinical cutoffs for CTQ-SF subscales, which may have resulted in some loss of information, although alternative specifications using the standardized CTQ-SF total score and the number of CA&N subtypes yielded broadly similar findings. In addition, the study assessed caregivers’ own CA&N but did not use child self-reports to measure whether the study children themselves experienced abuse or neglect. Future research incorporating richer measures across both generations would allow a more complete assessment of intergenerational pathways.

## 5 Conclusion

Using nine years of longitudinal data from rural western China, this study found that caregiver CA&N was associated with poorer cognitive and social-emotional development in the next generation, with cognitive disparities widening as children aged. Statistical mediation through parenting practices was limited: indirect associations were observed mainly for social-emotional outcomes and ran chiefly through neglectful parenting, with little evidence of mediation for cognitive outcomes. Associations did not differ significantly between parental and grandparental caregivers. Together, these findings extend evidence on the intergenerational correlates of CA&N to a longitudinal LMIC setting and point to neglectful parenting as one potentially modifiable pathway linking caregiver CA&N with children's social-emotional development.

## CRediT authorship contribution statement

**Qiannan Song:** Writing – review & editing, Writing – original draft, Methodology, Investigation, Formal analysis, Conceptualization. **Hao Gong:** Writing – review & editing, Investigation, Formal analysis, Data curation. **Mingchuan Liu:** Writing – review & editing, Investigation, Data curation. **Na Qiao:** Writing – review & editing, Investigation, Funding acquisition, Data curation. **Ai Yue:** Writing – review & editing, Validation, Supervision, Methodology, Funding acquisition, Conceptualization. **Yaojiang Shi:** Supervision, Resources, Funding acquisition.

## Ethics declaration

Informed consent to take part in the study and to publish the article was obtained from all participants or their legal representatives. Written informed consent was obtained from caregivers in Waves 0–3. In Wave 4, the consent process was embedded in the tablet-based questionnaire. At the beginning of each interview, trained enumerators read aloud a standardized informed consent statement displayed at the start of the questionnaire, covering the study purpose, what participation involved, confidentiality, and the voluntary nature of participation. Caregivers were then asked whether they agreed to participate on behalf of themselves and their child. The interview proceeded only after the caregiver provided verbal informed consent, and the consent process was audio-recorded in the tablet-based survey system. This approach was particularly suitable in the rural study setting, where some caregivers, especially grandparents, had limited literacy. Waves 0–2 were approved by the Stanford University Institutional Review Board (ID: 35921), Wave 3 by the Biological and Medical Ethics Review Committee of Minzu University of China (ID: ECMUC2020027CO), and Wave 4, including the verbal consent procedure, by the same committee (ID: ECMUC2023005CO). All data were anonymized, and no identifiable information about participants is reported. The privacy rights of participants have been observed.

This study was performed in compliance with relevant laws, regulatory frameworks and guidelines where the research took place. This study was approved by the Stanford University Institutional Review Board; Biological and Medical Ethics Review Committee of Minzu University of China. (Approval No. 35921 (Stanford, Waves 0–2); ECMUC2020027CO (Minzu, Wave 3); ECMUC2023005CO (Minzu, Wave 4))

## Declaration of generative AI and AI-assisted technologies in the manuscript preparation process

During the preparation of this work the authors used ChatGPT (OpenAI) in order to improve the language and readability of the manuscript. After using this tool, the authors reviewed and edited the content as needed and take full responsibility for the content of the published article.

## Funding

This project was supported by the Natural Science Foundation of Shaanxi [2024JC-ZDXM-05]; General Project of the 10.13039/501100017596Natural Science Basic Research Program of Shaanxi Province [2025JC-YBQN-1005]; General Project of the Philosophy and Social Sciences Research Special Program of Shaanxi Province [2025YB0311]; Youth Innovation Team Program of Shaanxi Universities, the Shaanxi Normal University Graduate Interdisciplinary Training Program [GERP-26-28]; HuPan Modou Foundation; and Key Laboratory of Modern Teaching Technology, Ministry of Education.

## Declaration of interest statement

The authors declared no potential conflicts of interest.

## Data Availability

Data will be made available on request.

## References

[bib1] Austin P.C. (2011). An introduction to propensity score methods for reducing the effects of confounding in observational studies. Multivariate Behavioral Research.

[bib2] Badenes-Ribera L., Georgieva S., Tomás J.M., Navarro-Pérez J.J. (2024). Internal consistency and test-retest reliability: A reliability generalization meta-analysis of the Childhood Trauma Questionnaire – Short Form (CTQ-SF). Child Abuse & Neglect.

[bib3] Bai Y., Liu B., Wang L., Li M., Zhang S., Dang R., Zheng L., Yang N., Song C. (2021). Parenting practices and cognitive development of preschool-aged children: Evidence from rural China. Journal of Child and Family Studies.

[bib4] Bai Y., Zheng L., Liu B., Yang N., Chen P. (2019). An empirical study on the status of rural parenting behavior and its influence in poor rural China. Journal of East China Normal University (Educational Sciences).

[bib5] Bernstein D.P., Fink L. (1998).

[bib6] Bernstein D.P., Stein J.A., Newcomb M.D., Walker E., Pogge D., Ahluvalia T., Stokes J., Handelsman L., Medrano M., Desmond D., Zule W. (2003). Development and validation of a brief screening version of the Childhood Trauma Questionnaire. Child Abuse & Neglect.

[bib7] Bian X.-Y., Squires J., Lu H.-M., Yi Y., Wang G.-W., Xu R.-C., Song W., Zhang J. (2021). Study on the norm and the reliability of the second edition of the Ages & Stages Questionnaires: Social-Emotional with a Chinese national sample. Chinese Journal of Child Health Care.

[bib8] Bowlby J. (1973).

[bib9] Chen D., Lin L., Li C., Chen W., Zhang Y., Ren Y., Guo V.Y. (2023). Maternal adverse childhood experiences and health-related quality of life in preschool children: A cross-sectional study. Child and Adolescent Psychiatry and Mental Health.

[bib10] Conger R.D., Donnellan M.B. (2007). An interactionist perspective on the socioeconomic context of human development. Annual Review of Psychology.

[bib11] Du Y., Kou J., Wang X., Xia L., Zou R.-H. (2006). A study on the Strengths and Difficulties Questionnaire (SDQ). Psychological Science.

[bib12] Dye H. (2018). The impact and long-term effects of childhood trauma. Journal of Human Behavior in the Social Environment.

[bib13] Fan W., Qian Y. (2015). Long-term health and socioeconomic consequences of early-life exposure to the 1959–1961 Chinese famine. Social Science Research.

[bib14] Folger A.T., Eismann E.A., Stephenson N.B., Shapiro R.A., Macaluso M., Brownrigg M.E., Gillespie R.J. (2018). Parental adverse childhood experiences and offspring development at 2 years of age. Pediatrics.

[bib15] Greene C.A., Haisley L., Wallace C., Ford J.D. (2020). Intergenerational effects of childhood maltreatment: A systematic review of the parenting practices of adult survivors of childhood abuse, neglect, and violence. Clinical Psychology Review.

[bib16] Hardt J., Rutter M. (2004). Validity of adult retrospective reports of adverse childhood experiences: Review of the evidence. Journal of Child Psychology and Psychiatry.

[bib17] He J., Zhong X., Gao Y., Xiong G., Yao S. (2019). Psychometric properties of the Chinese version of the Childhood Trauma Questionnaire-Short Form (CTQ-SF) among undergraduates and depressive patients. Child Abuse & Neglect.

[bib18] Herbert K., Xi Q., Feder G., Gilbert R., Powell C., Howarth E., Morris S. (2023). Child maltreatment and parental domestic violence and abuse, co-occurrence and the effect on lifetime outcomes in the Avon Longitudinal Study of Parents and Children (ALSPAC). SSM- Population Health.

[bib19] Laird N.M., Ware J.H. (1982). Random-effects models for longitudinal data. Biometrics.

[bib20] Lieberman A.F., Diaz M.A., Castro G., Oliver Bucio G. (2020).

[bib21] Liu J. (2025). Childhood and rural to urban migration in China: A tale of three villages. Children & Society.

[bib22] Lotto C.R., Altafim E.R.P., Linhares M.B.M. (2023). Maternal history of childhood adversities and later negative parenting: A systematic review. Trauma, Violence, & Abuse.

[bib24] Luo J., Peng X., Zong R., Yao K., Hu R., Du Q., Fang J., Zhu M. (2008). The status of care and nutrition of 774 left-behind children in rural areas in China. Public Health Reports.

[bib23] Luo S., Chen D., Li C., Lin L., Chen W., Ren Y., Zhang Y., Xing F., Guo V.Y. (2023). Maternal adverse childhood experiences and behavioral problems in preschool offspring: The mediation role of parenting styles. Child and Adolescent Psychiatry and Mental Health.

[bib25] MacKinnon D.P., Lockwood C.M., Hoffman J.M., West S.G., Sheets V. (2002). A comparison of methods to test mediation and other intervening variable effects. Psychological Methods.

[bib26] Madigan S., Plamondon A., Jenkins J.M. (2025). The long reach of adversity: Intermediary pathways from maternal adverse childhood experiences to child socio-emotional and cognitive outcomes. Journal of Child Psychology and Psychiatry.

[bib27] Main M., Hesse E., Greenberg M.T., Cicchetti D., Cummings E.M. (1990). Attachment in the preschool years: Theory, research, and intervention.

[bib28] McEwen C.A., Gregerson S.F. (2019). A critical assessment of the adverse childhood experiences study at 20 years. American Journal of Preventive Medicine.

[bib29] Mize T.D. (2022). sgmediation2: Sobel-Goodman tests of mediation in Stata. https://www.trentonmize.com/software/sgmediation2.

[bib30] Narayan A.J., Lieberman A.F., Masten A.S. (2021). Intergenerational transmission and prevention of adverse childhood experiences (ACEs). Clinical Psychology Review.

[bib31] Ongilio F.L., Gaspardo C.M., Linhares M.B.M. (2023). Maternal history of adversity and subsequent mother–child interactions at early ages: A systematic review. Trauma, Violence, & Abuse.

[bib32] Pan J., Yang W., Chen J., Xi W., Zhang H., Peng Y., Gu G., Zhang Y., Zhong Z., Jiao F., Qin R., Li J., Chu Y., Yang J., Wang W., Ma L., Teng H., Gao Y., Ying Y., Liu T. (2014). Development of neglect evaluation scale for primary school students aged 6–11 years old in rural areas of China. Chinese Journal of Preventive Medicine.

[bib33] Plant D.T., Jones F.W., Pariante C.M., Pawlby S. (2017). Association between maternal childhood trauma and offspring childhood psychopathology: Mediation analysis from the ALSPAC cohort. The British Journal of Psychiatry.

[bib34] Preacher K.J., Hayes A.F. (2004). SPSS and SAS procedures for estimating indirect effects in simple mediation models. Behavior Research Methods, Instruments, & Computers.

[bib35] Preacher K.J., Hayes A.F. (2008). Asymptotic and resampling strategies for assessing and comparing indirect effects in multiple mediator models. Behavior Research Methods.

[bib36] Prime H., Andrews K., Markwell A., Gonzalez A., Janus M., Tricco A.C., Bennett T., Atkinson L. (2023). Positive parenting and early childhood cognition: A systematic review and meta-analysis of randomized controlled trials. Clinical Child and Family Psychology Review.

[bib37] Pryce C.R., Pryce C.R., Martin R.D., Skuse D. (1995). Motherhood in human and nonhuman primates: Biosocial determinants.

[bib38] Racine N., Deneault A.-A., Thiemann R., Turgeon J., Zhu J., Cooke J.E., Madigan S. (2025). Intergenerational transmission of parent adverse childhood experiences to child outcomes: A systematic review and meta-analysis. Child Abuse & Neglect.

[bib39] Racine N., Plamondon A., Madigan S., McDonald S., Tough S. (2018). Maternal adverse childhood experiences and infant development. Pediatrics.

[bib40] Rosenbaum P.R., Rubin D.B. (1983). The central role of the propensity score in observational studies for causal effects. Biometrika.

[bib41] Sales W.B., Maranhão E.F., Ramalho C.S.T., Macêdo S.G.G.F., Souza G.F., Maciel Á.C.C. (2024). Early life circumstances and their impact on health in adulthood and later life: A systematic review. BMC Geriatrics.

[bib42] Savage L.-É., Tarabulsy G.M., Pearson J., Collin-Vézina D., Gagné L.-M. (2019). Maternal history of childhood maltreatment and later parenting behavior: A meta-analysis. Development and Psychopathology.

[bib43] Singer J.D., Willett J.B. (2003).

[bib44] Squires J., Bricker D., Twombly E. (2002).

[bib45] Stoltenborgh M., Bakermans-Kranenburg M.J., Alink L.R.A., van IJzendoorn M.H. (2015). The prevalence of child maltreatment across the globe: Review of a series of meta-analyses. Child Abuse Review.

[bib46] Swords L., Kennedy M., Spratt T. (2024). Pathways explaining the intergenerational effects of ACEs: The mediating roles of mothers' mental health and the quality of their relationships with their children. Journal of Applied Developmental Psychology.

[bib47] Sylvia S., Luo R., Zhong J., Dill S.-E., Medina A., Rozelle S. (2022). Passive versus active service delivery: Comparing the effects of two parenting interventions on early cognitive development in rural China. World Development.

[bib48] Tan C., Luo J., Zong R., Fu C., Zhang L., Mou J., Duan D. (2010). Nutrition knowledge, attitudes, behaviours and the influencing factors among non-parent caregivers of rural left-behind children under 7 years old in China. Public Health Nutrition.

[bib49] van IJzendoorn M.H., Bakermans-Kranenburg M.J., Coughlan B., Reijman S. (2020). Annual research review: Umbrella synthesis of meta-analyses on child maltreatment antecedents and interventions: Differential susceptibility perspective on risk and resilience. Journal of Child Psychology and Psychiatry.

[bib50] Wang L., Jiang D., Chen Y., Zhang S., Rozelle S. (2024). Paths of cognitive and social-emotional delays before age three in rural China: Predictive power on skills at preschool age. PLoS One.

[bib51] Wang L., Liang W., Zhang S., Jonsson L., Li M., Yu C., Sun Y., Ma Q., Bai Y., Abbey C., Luo R., Yue A., Rozelle S. (2019). Are infant/toddler developmental delays a problem across rural China?. Journal of Comparative Economics.

[bib52] Wang X., Yin G., Guo F., Hu H., Jiang Z., Li S., Shao Z., Wan Y. (2022). Associations of maternal adverse childhood experiences with behavioral problems in preschool children. Journal of Interpersonal Violence.

[bib53] Weistra S.R., van Bakel H.J.A., Mathijssen J.J.P. (2025). Adverse childhood experiences in parental history and how they relate to subsequent observed parent–child interaction: A systematic review. Child & Youth Care Forum.

[bib54] Xu P., Liu Z., Xu Y., Li T., Xu G., Xu X., Wang L., Yan Y., Xiao S., Li L., Zhang T., Yan J., Yu Y., Xu X., Wang Z., Wang B., Guo W., Huang Y. (2024). The prevalence and profiles of adverse childhood experiences and their associations with adult mental health outcomes in China: A cross-sectional study. Lancet Regional Health. Western Pacific.

[bib55] Yue A., Bai Y., Shi Y., Luo R., Rozelle S., Medina A., Sylvia S. (2020). Parental migration and early childhood development in rural China. Demography.

[bib56] Yue A., Song Q., Gao Y., Liang S., Zhang X., Zhang Y., Ding Y., Shi Y. (2024). Effect of parenting centers on caregiver mental health: Evidence from a large scale randomized controlled trial in rural China. China Economic Review.

[bib57] Zhang J., Cooke E., Wei X., Liu Y., Zheng Y. (2024). Intergenerational cascade processes from parental childhood adversity to child emotional and behavioral problems. Child Abuse & Neglect.

[bib58] Zhang L., Mersky J.P., Gruber A.M.H., Kim J.-Y. (2023). Intergenerational transmission of parental adverse childhood experiences and children's outcomes: A scoping review. Trauma, Violence, & Abuse.

[bib59] Zhang L., Mersky J.P., Lee C.P. (2023). Intergenerational pathways linking mothers' adverse childhood experiences and children's social-emotional problems. Child Maltreatment.

[bib60] Zhu J., Racine N., Tough S., Madigan S. (2025). Pathways of intergenerational risk: Examining the association between maternal adverse childhood experiences and child socio-emotional and behavioral concerns at 8 years of age. Child Maltreatment.

