# Supplementary material for: Caregiver childhood abuse and neglect and children's cognitive and social-emotional development: The role of parenting practices
Source: SSM Popul Health. 2026 Oct 15;36:101988. doi: 10.1016/j.ssmph.2026.101988 (PMC13698069; doi:10.1016/j.ssmph.2026.101988)
Supplement: Supplementary file 1 [file mmc1.docx]

Caregiver childhood abuse and neglect and children’s cognitive and social-emotional development: The role of parenting practices

**Supplementary material**

**Appendix I: Statistical Models**

**Appendix II: Supplementary Figures and Tables**

**Appendix I: Statistical Models**

**Mediation models**

To examine potential indirect pathways, we assessed whether parenting practices statistically mediated the associations between caregiver childhood abuse and neglect (CA&N) and children’s developmental outcomes. Because the data are observational, these analyses identify indirect associations rather than causal mediation (VanderWeele, 2016). Analyses were implemented in Stata using the user-written sgmediation2 module (Mize, 2022), an updated version of the sgmediation command originally developed by the UCLA Statistical Consulting Group, which conducts Sobel–Goodman tests of statistical mediation for linear regression models. Within the Baron and Kenny (1986) framework, the procedure fits three regressions—the outcome on the exposure (total association, path c), the mediator on the exposure (path a), and the outcome on both the exposure and the mediator (paths b and c′)—each adjusting for the covariates defined in Section 2.2.4 of the main text, an indicator of primary caregiver change, and the developmental outcome measured at the preceding wave, and computes the indirect association as the product of coefficients a×b (Sobel, 1982; MacKinnon et al., 2002). Bootstrap standard errors and bias-corrected 95% confidence intervals were obtained from 1,000 non-parametric bootstrap replications, following the resampling recommendations of Preacher and Hayes (2004, 2008).

**Heterogeneity models**

To assess heterogeneity by caregiver type, we estimated the main mixed-effects model (Equation 1 in the main text), without the CA&N × wave interaction term, separately within two subsamples defined by the type of the Wave 4 reference caregiver—children cared for by parents and children cared for by grandparents:

$Y_{it}=\beta_{0}+\beta_{1}{c\_wave}_{it}+\beta_{2}{c\_wave}_{it}^{2}+\beta_{3}{CAN}_{i}+\gamma X_{i}+u_{0i}+u_{1i}{c\_wave}_{it}+\varepsilon_{it}$, estimated separately for parent- and grandparent-caregiver subsamples.

As in Equation 1, the coefficient $\beta_{3}$ captures the association between caregiver CA&N and the developmental outcome within each caregiver-type subsample. The covariate vector comprises the covariates listed in the note to Table 5 of the main text. Differences between the parent- and grandparent-specific CA&N coefficients were evaluated using tests of coefficient equality, as reported in the Difference columns of Table 5 in the main text; because these tests did not reach conventional significance, the subgroup contrasts are interpreted as suggestive rather than definitive.

**Appendix II: Supplementary Figures and Tables**

**Figure S1.** Longitudinal study timeline: sample sizes, retention, and key variables collected.

**Figure S2.** Distribution of propensity scores by caregiver CA&N exposure (Exposed vs. Unexposed).

**Figure S3.** Love plot of covariate-specific standardized percentage bias (standardized mean difference × 100) before and after one-to-one nearest-neighbor propensity score matching (caliper = 0.05). Red circles denote the unmatched sample and blue diamonds the matched sample; after matching, the absolute standardized biases for all covariates fell below the conventional 10% threshold.

(a) Cognitive outcomes	(b) Social-emotional outcomes

**Figure S4.** Predicted trajectories of children’s cognitive and social-emotional outcomes by caregiver CA&N exposure. Lines show predicted values from the full-sample mixed-effects models in Table 2; shaded areas show 95% confidence intervals.

**(a)** Full sample: PN and Cognitive **(b)** Consistent-caregiver subsample: PN and Cognitive

**(c)** Full sample: PN and Social-emotional **(d)** Consistent-caregiver subsample: PN and

Social-emotional

**(e)** Full sample: EN and Cognitive **(f)** Consistent-caregiver subsample: EN and Cognitive

**(g)** Full sample: EN and Social-emotional	**(h)** Consistent-caregiver subsample: EN and	Social-emotional

**Figure S5.** Predicted trajectories of children’s cognitive and social-emotional outcomes by caregiver physical neglect (PN) and emotional neglect (EN) exposure. Lines show predicted values from the mixed-effects models in Table 3; shaded areas show 95% confidence intervals.

**Table S1**

Dose–response robustness: longitudinal associations between continuous measures of caregiver CA&N and children’s cognitive and social-emotional outcomes.

| **Variables** | **Standardized cognitive scores** | | | **Standardized social-emotional scores** | | |
| --- | --- | --- | --- | --- | --- | --- |
|  | **β (SE)** | **p-value** | **95% CI** | **β (SE)** | **p-value** | **95% CI** |
| **Panel A. Exposure = No. of CA&N subtypes (0–5)** | | | | | | |
| No. of CA&N subtypes | –0.08*** (0.02) | < 0.001 | –0.12 to –0.04 | –0.15*** (0.02) | < 0.001 | –0.19 to –0.12 |
| Wave (centered) | 0.02 (0.01) | 0.142 | –0.01 to 0.04 | 0.01 (0.01) | 0.282 | –0.01 to 0.03 |
| Wave (centered)² | –0.01* (0.01) | 0.038 | –0.03 to 0.00 | 0.04*** (0.01) | < 0.001 | 0.02 to 0.05 |
| CA&N subtypes × Wave | –0.02* (0.01) | 0.037 | –0.04 to 0.00 | –0.02 (0.01) | 0.054 | –0.04 to 0.00 |
| Observations | 5,381 | | | 5,381 | | |
| **Panel B. Exposure = CTQ-SF total score (standardized)** | | | | | | |
| CTQ-SF total score (z) | –0.08*** (0.02) | < 0.001 | –0.12 to –0.04 | –0.17*** (0.02) | < 0.001 | –0.20 to –0.13 |
| Wave (centered) | 0.00 (0.01) | 0.831 | –0.02 to 0.02 | 0.00 (0.01) | 0.862 | –0.02 to 0.02 |
| Wave (centered)² | –0.01* (0.01) | 0.038 | –0.03 to 0.00 | 0.04*** (0.01) | < 0.001 | 0.02 to 0.05 |
| CTQ-SF score × Wave | –0.01 (0.01) | 0.315 | –0.03 to 0.01 | –0.02* (0.01) | 0.013 | –0.04 to 0.00 |
| Observations | 5,381 | | | 5,381 | | |
| Controls | YES | | | YES | | |

*Note.* β, standardized beta; SE, standard error; CI, confidence interval; CA&N, childhood abuse and neglect; CTQ-SF, Childhood Trauma Questionnaire-Short Form; Wave (centered), survey wave centered at the full-sample mean. In Panel A, the exposure is the number of CA&N subtypes above the clinical cutoffs (range 0–5); in Panel B, the exposure is the standardized CTQ-SF total score. Models control for child sex, birth order, left-behind status, whether the caregiver is the mother, maternal age at delivery, caregiver education, household assets, original trial arm, and primary caregiver change, replacing only the binary CA&N indicator with the continuous exposure metric. The number of observations (5,381) is smaller than in the main analysis (5,575) because continuous CTQ-SF scores could not be reconstructed for 58 children (194 child-wave observations) whose Wave 4 assessment was not retained in the analytic sample. Both continuous specifications reproduce the negative associations found with the binary indicator in Table 2, confirming that the findings are not an artifact of dichotomization. * p < 0.05, ** p < 0.01, *** p < 0.001.

**Table S2**

Robustness to village-level clustering.

| **Variables** | **Cognitive main** | **Cognitive 3-level** | **Cognitive vce (village)** | **Social-emotional main** | **Social-emotional 3-level** | **Social-emotional vce (village)** |
| --- | --- | --- | --- | --- | --- | --- |
| Caregiver CA&N (= 1; Ref = 0) | –0.12** (0.04) | –0.12** (0.04) | –0.12** (0.04) | –0.21*** (0.04) | –0.20*** (0.04) | –0.21*** (0.03) |
| Wave (centered) | 0.02* (0.01) | 0.02* (0.01) | 0.02 (0.01) | 0.01 (0.01) | 0.01 (0.01) | 0.01 (0.01) |
| Wave (centered)² | –0.01 (0.01) | –0.01 (0.01) | –0.01 (0.01) | 0.04*** (0.01) | 0.04*** (0.01) | 0.04*** (0.01) |
| Caregiver CA&N × Wave | –0.05** (0.02) | –0.05** (0.02) | –0.05* (0.02) | –0.02 (0.02) | –0.02 (0.02) | –0.02 (0.02) |
| Observations | 5,575 | 5,575 | 5,575 | 5,575 | 5,575 | 5,575 |

*Note.* Standard errors in parentheses. CA&N, childhood abuse and neglect; Ref, reference group. Models control for child sex, birth order, left-behind status, whether the caregiver is the mother, maternal age at delivery, caregiver education, household assets, original trial arm, and primary caregiver change. Main = two-level model as in Table 2; 3-level adds village random intercepts; vce (village) clusters standard errors at the village level. Village-level intraclass correlation coefficient (ICC) from unconditional models: 0.03 (cognitive), 0.01 (social-emotional). * p < 0.05, ** p < 0.01, *** p < 0.001.

| **Table S3**  Longitudinal associations between caregiver CA&N and children’s cognitive and social-emotional outcomes (from 4 months to 10 years of age): full sample and control group sample. | | | | | | | | | | | | |
| --- | --- | --- | --- | --- | --- | --- | --- | --- | --- | --- | --- | --- |
| Variables | Standardized cognitive scores | | | | | | Standardized social-emotional scores | | | | | |
|  | Full sample (5,575 child-wave observations) | | | Control group sample (2,647 child-wave observations) | | | Full sample (5,575 child-wave observations) | | | Control group sample (2,647 child-wave observations) | | |
|  | β (SE) | p-value | 95% CI | β (SE) | p-value | 95% CI | β (SE) | p-value | 95% CI | β (SE) | p-value | 95% CI |
| Caregiver CA&N (= 1; Ref = 0) | –0.12** (0.04) | 0.002 | –0.20 to –0.04 | –0.11 (0.06) | 0.063 | –0.22 to 0.01 | –0.21*** (0.04) | < 0.001 | –0.28 to –0.14 | –0.17*** (0.05) | < 0.001 | –0.27 to –0.07 |
| Wave (centered) | 0.02* (0.01) | 0.046 | 0.00 to 0.05 | 0.01 (0.02) | 0.499 | –0.02 to 0.04 | 0.01 (0.01) | 0.641 | –0.02 to 0.03 | 0.01 (0.02) | 0.625 | –0.03 to 0.04 |
| Wave (centered)^2^ | –0.01 (0.01) | 0.087 | –0.02 to 0.00 | 0.00 (0.01) | 0.614 | –0.01 to 0.02 | 0.04*** (0.01) | < 0.001 | 0.02 to 0.05 | 0.03*** (0.01) | < 0.001 | 0.01 to 0.05 |
| Caregiver CA&N × Wave | –0.05** (0.02) | 0.007 | –0.08 to –0.01 | –0.05* (0.02) | 0.038 | –0.10 to 0.00 | –0.02 (0.02) | 0.368 | –0.05 to 0.02 | –0.03 (0.03) | 0.199 | –0.08 to 0.02 |
| Primary caregiver changed (= 1) | 0.09* (0.04) | 0.026 | 0.01 to 0.18 | 0.07 (0.06) | 0.232 | –0.05 to 0.19 | 0.14*** (0.04) | < 0.001 | 0.06 to 0.21 | 0.12* (0.05) | 0.023 | 0.02 to 0.22 |
| Constant | –0.17 (0.15) | 0.273 | –0.47 to 0.13 | –0.13 (0.22) | 0.558 | –0.56 to 0.30 | –0.04 (0.14) | 0.762 | –0.32 to 0.23 | 0.03 (0.19) | 0.869 | –0.35 to 0.41 |
| Controls | YES |  |  | YES |  |  | YES |  |  | YES |  |  |
| *Note.* Standard errors in parentheses. Values of 1 indicate caregiver CA&N exposure, and 0 indicate no exposure. CA&N, childhood abuse and neglect; Ref, reference group; β, standardized beta; SE, standard error; CI, confidence interval; Wave (centered), survey wave centered at the full-sample mean in both samples. Models control for child sex, birth order, left-behind status, whether the caregiver is the mother, maternal age at delivery, caregiver education, household assets, and primary caregiver change; full-sample models additionally control for original trial arm, which is constant within the control group sample and therefore omitted from the control-group models. Control group sample includes children from families assigned to the control arm of the original parenting trial. ** p < 0.05, ** p < 0.01, *** p < 0.001.* | | | | | | | | | | | | |

| **Table S4**  Longitudinal associations between caregiver CA&N and children’s cognitive and social-emotional outcomes: conventional vs. propensity score–matched models. | | | | | | | | | | | | |
| --- | --- | --- | --- | --- | --- | --- | --- | --- | --- | --- | --- | --- |
| Variables | Standardized cognitive scores | | | | | | Standardized social-emotional scores | | | | | |
|  | Conventional | | | Propensity score–matched models | | | Conventional | | | Propensity score–matched models | | |
|  | β (SE) | p-value | 95% CI | β (SE) | p-value | 95% CI | β (SE) | p-value | 95% CI | β (SE) | p-value | 95% CI |
| Caregiver CA&N (= 1; Ref = 0) | –0.12** (0.04) | 0.002 | –0.20 to –0.04 | –0.15** (0.05) | 0.002 | –0.24 to –0.05 | –0.21*** (0.04) | < 0.001 | –0.28 to –0.14 | –0.21*** (0.04) | < 0.001 | –0.29 to –0.12 |
| Wave (centered) | 0.02* (0.01) | 0.046 | 0.00 to 0.05 | 0.03* (0.02) | 0.047 | 0.00 to 0.07 | 0.01 (0.01) | 0.641 | –0.02 to 0.03 | 0.01 (0.02) | 0.425 | –0.02 to 0.04 |
| Wave (centered)^2^ | –0.01 (0.01) | 0.087 | –0.02 to 0.00 | 0.00 (0.01) | 0.696 | –0.02 to 0.02 | 0.04*** (0.01) | < 0.001 | 0.02 to 0.05 | 0.03** (0.01) | 0.001 | 0.01 to 0.05 |
| Caregiver CA&N × Wave | –0.05** (0.02) | 0.007 | –0.08 to –0.01 | –0.06* (0.02) | 0.010 | –0.10 to –0.01 | –0.02 (0.02) | 0.368 | –0.05 to 0.02 | –0.02 (0.02) | 0.289 | –0.06 to 0.02 |
| Primary caregiver changed (= 1) | 0.09* (0.04) | 0.026 | 0.01 to 0.18 | 0.09 (0.05) | 0.050 | 0.00 to 0.19 | 0.14*** (0.04) | < 0.001 | 0.06 to 0.21 | 0.16*** (0.05) | < 0.001 | 0.07 to 0.25 |
| Constant | –0.17 (0.15) | 0.273 | –0.47 to 0.13 | –0.27 (0.20) | 0.177 | –0.65 to 0.12 | –0.04 (0.14) | 0.762 | –0.32 to 0.23 | –0.04 (0.18) | 0.804 | –0.39 to 0.30 |
| Controls | YES |  |  | YES |  |  | YES |  |  | YES |  |  |
| *Note.* Standard errors in parentheses. Values of 1 indicate caregiver CA&N exposure, and 0 indicate no exposure. CA&N, childhood abuse and neglect; Ref, reference group; β, standardized beta; SE, standard error; CI, confidence interval; Wave (centered), survey wave centered at the full-sample mean in both samples. Models control for child sex, birth order, left-behind status, whether the caregiver is the mother, maternal age at delivery, caregiver education, household assets, original trial arm, and primary caregiver change. Conventional = mixed-effects model as in Table 2; Propensity score–matched = mixed-effects model estimated in the matched sample using matching weights from one-to-one nearest-neighbor propensity score matching (caliper = 0.05), as described in Section 2.3 of the main text. Matching was performed with replacement; the matched sample comprised 627 exposed children and 308 matched unexposed children (4,168 child-wave observations), whereas the conventional models used the full sample (5,575 child-wave observations). Primary caregiver changed (1 = changed at least once across waves, 0 = no change). * p < 0.05, ** p < 0.01, *** p < 0.001. | | | | | | | | | | | | |

**References**

Baron, R. M., & Kenny, D. A. (1986). The moderator–mediator variable distinction in social psychological research: Conceptual, strategic, and statistical considerations. Journal of Personality and Social Psychology, 51(6), 1173–1182. https://doi.org/10.1037/0022-3514.51.6.1173.

MacKinnon, D. P., Lockwood, C. M., Hoffman, J. M., West, S. G., & Sheets, V. (2002). A comparison of methods to test mediation and other intervening variable effects. Psychological Methods, 7(1), 83–104. https://doi.org/10.1037/1082-989X.7.1.83.

Mize, T. D. (2022). sgmediation2: Sobel-Goodman tests of mediation in Stata [Computer software]. <https://www.trentonmize.com/software/sgmediation2>.

Preacher, K. J., & Hayes, A. F. (2004). SPSS and SAS procedures for estimating indirect effects in simple mediation models. Behavior Research Methods, Instruments, & Computers, 36(4), 717–731. https://doi.org/10.3758/BF03206553.

Preacher, K. J., & Hayes, A. F. (2008). Asymptotic and resampling strategies for assessing and comparing indirect effects in multiple mediator models. Behavior Research Methods, 40(3), 879–891. https://doi.org/10.3758/BRM.40.3.879.

Sobel, M. E. (1982). Asymptotic confidence intervals for indirect effects in structural equation models. Sociological Methodology, 13, 290–312. <https://doi.org/10.2307/270723>.

VanderWeele, T. J. (2016). Mediation analysis: A practitioner’s guide. Annual Review of Public Health, 37, 17–32. https://doi.org/10.1146/annurev-publhealth-032315-021402.
